# Chicago Society of Physicians and Surgeons

**Published:** 1874-02-01

**Authors:** 


### CHICAGO SOCIETY OF PHYSICIANS AND SURGEONS.

AT the regular meeting of the Society, held on Monday evening, January 12th, the following memoranda of cases in the Surgical Ward of Mercy Hospital was presented frōm the Comittee on Hospital Reports:

CASE I.—*Necrosis of femur; operation, after application of elastic bandage.*—The patient, a boy, twelve years of age, had had necrosis in lower half of femur for two years; general health good. The limb was bandaged firmly, from the toes to the upper third of thigh, and tied around at top of bandage, firmly, with five coils of heavy rubber tubing of about half an inch exterior diameter. The elastic bandage was then removed, the limb appearing perfectly blanched. Prof. Andrews then proceeded to operate, removing several small sequestra without any appreciable loss of blood.

The elastic ligature was now removed, causing, for a few moments some oozing of blood from the wound. At the same time a somewhat singular phenomenon occurred, which was the appearance of a bright scarlet flush, from the toes up to the line of the ligatures, about as bright as in scarlatina. This lasted about ten minutes, and then faded away to the natural color of the skin.

CASE II.—*Amputation of middle finger ot metacarpal articulation.*—Desiring to avoid hæmorrhage, and not having the elastic bandage or tubing on hand, the limb was raised to a perpendicular position, and the blood pressed out by vigorous stroking of the limb downward. The tourniquet was then applied, and the amputation performed with but little loss of blood.

CASE III.—*Fractures of Pelvis.*—A case of fracture through the middle of the crest of left ilium downward into the ischiatic notch; patient recovering without any appearance of dangerous symptoms. No especial treatment, but rest in a recumbent position.

Another case of fracture of pelvis was recently discharged from the hospital, recovery having taken place without any unfavorable symptoms supervening. The experience of Prof. Andrews goes to show that these cases usually make good recoveries when the viscera are not wounded.

CASE IV.—*Pott's Disease of Neck.* The case is being treated by the application of a brass armor, so to speak, moulded from a plaster cast of the neck and shoulders, taken with the head strongly flexed backwards.

CASE V.—*Empyema from gun-shot wound treated by drainage tubing.*—Is gaining flesh and strength, and in fair way to recover. Suppurative discharge is suppressed by daily injections of sol. carbolic acid, ten grains to the ounce.

*Strictures of the Urethra.—Some cases under treatment.*—Prof. Andrews usually treats by internal section, and subsequent frequent use of bougie. The most convenient instrument for very tight strictures is probably Maisoneuve's stricture-cutter. Owing to its delicacy of construction, however, it is constantly getting out of repair.

The clinical lecture on Delirium Tremens, which we print in another department of the present number of THE EXAMINER, was also presented to the Society from this Comittee, and called forth considerable discussion from the members.

Dr. Andrews remarked that he had just operated upon two additional cases of necrosis, the elastic bandage being applied as before described, and with the effect of completely preventing hæmorrhage; and, also, much facilitating the operation, by the bloodless condition of the parts.

Dr. Owens brought in, for the inspection of the Society, a boy about fifteen years of age, whose left arm had been amputated at the shoulder by the passing over it of a car-wheel. Some loose flaps of skin, and a projecting edge of the clavicle were trimmed off, and the arm dressed—the car-wheel making as clean and smooth an amputation as a surgeon could have done. No hæmorrhage took place after the accident, and the parts healed rapidly, leaving a good stump.

F. H. D.

---

TO SUBSCRIBERS.—During the past month, bills for the current year's subscription have been sent out to all who had failed to send in their renewals. Most of these have been responded to with gratifying promptness, and with a large list of new subscribers. THE EXAMINER enters upon another year of assured success and prosperity. THE EXAMINER has never been published as a speculation, or as a money-making enterprise. All the income received from it is spent upon it, and, generally, somewhat more. Just as fast, therefore, as our subscription list increases, we are enabled to enlarge and improve the character of our issues. If our subscription list was double, we should double the size of our issues. There is no reason why this should not be done. It is a matter of personal interest to every one of our friends and subscribers. Let each one but try a little and obtain a few new subscribers for us in his neighborhood, and it will quickly be accomplished.

F. H. D.

OBITUARY. — DR. S. W. BUTLER, widely known to the profession as the editor and proprietor of the *Philadelphia Med. and Surg. Reporter*, died at his residence, in Philadelphia, on the 6th of Jan., of phthisis pulmonalis.

For over twenty years Dr. Butler had been engaged in editorial and literary work, in connection with the *Medical Reporter*, the *Half-Yearly Compendium*, and the other publications of his office.

The *Medical Reporter*, under his management, has become one of the leading and most successful periodicals of the country.

These publications will be continued, under the charge of Dr. D. G. Brinton, who has been for some time connected with, and assisting in, their management.

